# Detection of Structural Vibration with High-Rate Precise Point Positioning: Case Study Results Based on 100 Hz Multi-GNSS Observables and Shake-Table Simulation

**DOI:** 10.3390/s19224832

**Published:** 2019-11-06

**Authors:** Jacek Paziewski, Rafal Sieradzki, Radoslaw Baryla

**Affiliations:** Institute of Geodesy, University of Warmia and Mazury in Olsztyn, Oczapowskiego 1 st., 10-719 Olsztyn, Poland; rafal.sieradzki@uwm.edu.pl (R.S.); baryla@uwm.edu.pl (R.B.)

**Keywords:** precise point positioning, PPP, GNSS, structural health monitoring, displacements, high-rate observables

## Abstract

This contribution presents and assesses the methodology aiming at the characterization of the structural vibrations with high-rate GNSS measurements. As commonly employed precise point positioning (PPP) based on ionosphere-free linear combination of undifferenced signals may not meet the high requirements in terms of displacement precision, a modified processing strategy has been proposed. The algorithms were implemented in the own-developed GNSS processing software and validated using the designed experiment. For this purpose, we have set up a field experiment taking advantage of the prototype shake-table, which simulated the dynamic horizontal displacements of the GNSS antenna. The device ensured a periodic motion of the antenna with modifiable characteristics, namely amplitude and frequency. In this experiment, we have set the amplitudes from 1.5 to 9 mm and the frequency to 3.80 Hz. As a dataset, we have used 100 Hz GPS, Galileo, and BDS measurements. The results confirmed a high applicability of the enhanced PPP processing strategy for precise displacement detection. Specifically, it was feasible to obtain the dynamic displacements with precision at the level of millimeters. The differences between the PPP-derived amplitude and the true amplitude of the simulated displacements were in the range of 0.5–1.3 mm, whereas the difference between the detected and benchmark frequency did not exceed 0.026 Hz. Hence, the proposed methodology allows meeting the specific demands of structural displacement monitoring.

## 1. Introduction

Thanks to the development of processing algorithms and the observation collection hardware, GNSS technology is currently successfully employed for seismic studies, geodynamics, and deformation monitoring, as well as for the detection of dynamic displacements including structural health monitoring (SHM) [1,2,3]. The advances in high-rate GPS data processing for seismological studies led to labelling this domain as GPS seismology [4]. Now, GNSS technology is considered as a reliable source of information on the health of various structures such as bridges or tall buildings, which are under the influence of external forces and geohazard phenomena [5,6]. The GNSS positioning methods used for the characterization of the structural vibrations ought to satisfy the specific demands in terms of precision and reliability. Furthermore, on the contrary to geodynamic and seismic studies, in structural monitoring, it is required to employ observations with as high a temporal resolution as possible addressing the frequency of the structure vibration. Such data allow the investigation of the structure response in the broad spectrum of the frequency domain and the characterization of the structure under different ambient excitations [7,8]. Hence, GNSS measurements not only enable the engineers to evaluate of the impact of the external forces on the structures, but also support numerical models of the constructions.

Among a number of GNSS processing methods, there are few that may meet the specific demands of structural monitoring. These include a relative approach based on double-differenced data, direct displacement detection methods, and the absolute method—precise point positioning (PPP)—performed with undifferenced phase and code measurements [9,10]. GNSS relative positioning with phase ambiguities fixed to integer values guarantees a reliable and precise solution. As the most mature method from the list, the relative positioning is currently considered as the most precise method of kinematic coordinate estimation [11,12]. Up to now, a number of publications have shown high performance of the relative mode in seismic and SHM studies [13,14,15]. Researchers reported, for example, that the relative processing strategy supported with the external sensors such as accelerometers may provide the characteristics of the seismically-induced motion or structural vibration with a broad spectrum of the frequency and amplitude [16]. As confirmed in the works of [17,18,19], the real time solution, represented in this context by the real-time kinematics (RTK) method, is highly applicable to high-rise building and bridge monitoring. On the other hand, such an approach is still characterized with several limitations, from which the most important is the necessity of providing stable external reference stations. Thus, a potential malfunctioning of these sites would directly deteriorate or even spoil the solution. Moreover, in this case, the positioning accuracy decreases with the increasing length of the baselines.

The approaches that may address the aforementioned constraints are the direct measurement methods. The approaches such as VADASE [20], the phase residual method (PRM) [21], or the signal processing method (SPM) [9] provide displacements or velocities instead of coordinate time series in a specified reference frame. Such methods have to be considered as relative, because they illustrate only the variation of parameters in the time domain. The advantages of these methods are simple implementation to a real-time mode and an opportunity to process multi-constellation signals. Apart from these, recent contributions have also confirmed a high usability of PPP in both SHM and seismic studies. In this field, we observe a clear benefit from the application of multi-frequency and multi-constellation signals [22]. On the other hand, the PPP method requires support from the external precise products such as satellite clock corrections and orbits, as well as the application of the dedicated processing methodology to address the requirements of the high-rate detection of sub-centimeter displacements. At this point, we should acknowledge several studies that importantly contributed to the subject, such as the works of [23,24,25], but still we may identify the perspectives for further advances, especially in terms of the precision and the convergence time of the solution.

This paper is the extended version of the presentation given at the 4th Joint International Symposium on Deformation Monitoring (JISDM2019), which was held on 15–17 May 2019 in Athens, Greece. The goal of this contribution is to present the processing strategy based on the precise point positioning method, which is suitable for the high-rate and high-precision displacement detection. For validation purposes, we set up a field experiment taking advantage of the in-house made shake-table, which artificially induces dynamic displacements of the GNSS antenna. The device was designed to ensure a periodic motion of the antenna with the selected amplitude and frequency. The tests were conducted using GPS + Galileo + BDS data with a 100 Hz sampling rate. At this point, it should be noted that the high-rate PPP positioning is not supported by any commercial software. Hence, all computations were performed with own-developed software with implemented high-rate PPP processing algorithms [26].

This contribution is organized as follows. In the next section, we briefly present the methodology of the high-rate PPP. The following parts describe the experiment design and discuss the results. The last section is devoted to conclusions and perspectives.

## 2. Methodology

### 2.1. Multi-GNSS PPP Functional and Stochastic Model

Precise point positioning may provide displacements of the monitoring point with respect to a global reference frame using a single GNSS receiver. In contrast to the relative approach, in this case, the reference frame is defined by the satellite orbits and clocks [27,28]. Recent advances in algorithms, which led to PPP with resolved integer ambiguities, predestine this method to compete with the relative approach in terms of accuracy of the solution [23,29]. In this field, we should acknowledge methods such as the decoupled clock model or integer recovery clocks [30,31], as well as the method that takes advantage of uncalibrated hardware delays [32].

The starting point of the PPP methodology applied in this work is the equations of the ionosphere-free linear combination (IF-LC) of phase and pseudorange observations. Their basis lies in undifferenced phase and code observations, which, for frequency *n*, can be written as follows:

(1) $$\lambda_{n}\varphi_{l,n}^{i}=\rho_{l}^{i}+c\left(t_{l}-t^{i}\right)+\lambda_{n}\left(b_{l,n}-b_{n}^{i}\right)+\alpha_{l}^{i}ZTD_{l}-I_{l,n}^{i}+\lambda_{n}N_{l,n}^{i}+\epsilon_{l,\varphi ,n}^{i},$$

(2) $$P_{l,n}^{i}=\rho_{l}^{i}+c\left(t_{l}-t^{i}\right)+\left(d_{l,n}-d_{n}^{i}\right)+\alpha_{l}^{i}ZTD_{l}+I_{l,n}^{i}+\epsilon_{l,P,n}^{i},$$

where

λ is the wavelength on selected frequency signal;
φ is the phase observable in cycles;
P is the pseudorange in meters;
*l* and *i* represent station and satellite, respectively;
ρ is the geometric range between satellite and station;
$t_{l}$ and $t^{i}$ are the receiver and satellite clock corrections in seconds, respectively;
c is the speed of light in meters per second;
$b_{l}$ and $b^{i}$ denote the frequency-dependent receiver and satellite phase delays in cycles, respectively, which include initial and hardware phase biases;
$d_{l}$ and $d^{i}$ are the frequency-dependent receiver and satellite code biases in meters, respectively;
*α* refers to the troposphere mapping function coefficient;
*ZTD* denotes the zenith tropospheric delay;
I denotes ionospheric delay;
N is the phase ambiguity term;
ϵ denotes the observational noise.

Taking advantage of dual-frequency observations, we can form the ionosphere-free linear combination of phase and pseudorange signals [28] and eliminate the first order of the ionospheric delay as follows:

(3) $$\Phi_{l,IF}^{i}=\left(f_{1}^{2}\Phi_{l,1}^{i}-f_{2}^{2}\Phi_{l,2}^{i}\right)/\left(f_{1}^{2}-f_{2}^{2}\right),$$

(4) $$P_{l,IF}^{i}=\left(f_{1}^{2}P_{l,1}^{i}-f_{2}^{2}P_{l,2}^{i}\right)/\left(f_{1}^{2}-f_{2}^{2}\right),$$

where Φ is the phase observable in the units of meters corresponding to a particular frequency or combination; $f_{1}$, $f_{1}$ denote selected frequencies for each constellation (e.g., L1 and L2, E1 and E5a, and B1 and B2 in the case of GPS, Galileo, and BDS constellations).

*Functional model*

When we integrate multi-constellation observables, we are obliged to account for the time scale inter-system bias. This can be done by selecting one of the GNSS system time scales as the pivot one and estimating the inter-constellation time difference. The other possibility is to parameterize the receiver clock correction combined with receiver hardware delays individually for each GNSS system. In general, the researchers reported comparable performance of both approaches [33]. Because the latter strategy was employed in this case, the applied multi-constellation PPP functional model is given as follows (Equations (5)–(10)). The equations are derived for multi-constellation signals using superscripts G, E, and C for GPS, Galileo, and BDS, respectively.

(5) $$\Phi_{l,IF}^{i}=\rho_{l}^{i}+c{\bar{t}}_{l}^{G}+\alpha_{l}^{i}ZTD_{l}+B_{l,IF}^{i}+\epsilon_{l,\varphi ,IF}^{i},$$

(6) $$P_{l,IF}^{i}=\rho_{l}^{i}+c{\bar{t}}_{l}^{G}+\alpha_{l}^{i}ZTD_{l}+\epsilon_{l,P,IF}^{i},$$

(7) $$\Phi_{l,IF}^{j}=\rho_{l}^{j}+c{\bar{t}}_{l}^{E}+\alpha_{l}^{j}ZTD_{l}+B_{l,IF}^{j}+\epsilon_{l,\varphi ,IF}^{j},$$

(8) $$P_{l,IF}^{j}=\rho_{l}^{j}+c{\bar{t}}_{l}^{E}+\alpha_{l}^{j}ZTD_{l}+\epsilon_{l,P,IF}^{j},$$

(9) $$\Phi_{l,IF}^{k}=\rho_{l}^{k}+c{\bar{t}}_{l}^{C}+\alpha_{l}^{k}ZTD_{l}+B_{l,IF}^{k}+\epsilon_{l,\varphi ,IF}^{k},$$

(10) $$P_{l,IF}^{k}=\rho_{l}^{k}+c{\bar{t}}_{l}^{C}+\alpha_{l}^{k}ZTD_{l}+\epsilon_{l,P,IF}^{k},$$

where; *i*, *j*, *k* denote selected satellites of GPS, Galileo, and BDS constellations, respectively; $\left\{{\bar{t}}_{l}^{G},\;{\bar{t}}_{l}^{E},\;{\bar{t}}_{l}^{C}\right\}$ are clock corrections of receiver (*l*) combined with the receiver hardware delays corresponding to selected constellation; $\left\{B_{l,IF}^{i},\;B_{l,IF}^{j},\;B_{l,IF}^{k}\right\}$ denote ionosphere-free non-integer parameters including carrier-phase ambiguity terms coupled with the satellite and receiver phase biases.

As the approximate position of the antenna is known, this can be constrained with an appropriate a priori weight in the processing, providing faster convergence. The a priori variances that constrain the station coordinates correspond to the expected maximal displacements of the GNSS antenna. Other details of the employed PPP processing strategy, including correction models, are given in Table 1.

*Stochastic Model of Observables*

For the parameter estimation, we used a sequential least squares adjustment; hence, appropriate stochastic modelling is of high interest. Because the linear combination of original GNSS signals is used, their variances are derived using the law of random error propagation. Taking advantage of GPS L1/L2 frequencies and a priori phase signal variances, the variance of the ionosphere-free linear combination reads as follows:

(11) $$\sigma_{\varphi_{IF}}^{2,G}={\left(f_{1}^{2}/f_{1}^{2}-f_{2}^{2}\right)}^{2}\sigma_{\varphi_{1}}^{2,G}+{\left(f_{2}^{2}/f_{1}^{2}-f_{2}^{2}\right)}^{2}\sigma_{\varphi_{2}}^{2,G},$$

where $\sigma_{\varphi_{1}}^{G}$ and $\sigma_{\varphi_{2}}^{G}$ are the a priori variances of GPS L1 and L2 phase signals, respectively. Assuming that variances for L1 and L2 are equal for simplification, the variance of the *IF* combination reads as follows:

(12) $$\sigma_{\varphi_{IF}}^{2,G}\approx 8.9\cdot \sigma_{\varphi_{1}}^{2,G}.$$

Correspondingly, the variances for other systems and for code pseudoranges are obtained. According to Equation (12), we may expect the noise of combined observables to be a factor-three higher with respect to original observations. The weighting scheme applied for particular slant observables takes into account the initial variance of the IF signal and an elevation dependent function. Such derived values are placed at the diagonal of the variance matrix of observables. The matrix assumes no correlation between the frequencies and the types of measurement (code vs. phase). The a priori coordinates constraining is performed by the introduction of pseudo-observables to the observational model with corresponding weights computed as the inversion of variances of the constrained parameters.

At this point, we should acknowledge the studies on the application of advanced stochastic models for high-rate positioning. In the works of [7,34,35], the authors justified taking into account a cross correlation between dual-frequency signals and a time correlation. Shu et al. [35] reported a much higher time correlation of high-rate code observables than phase observables. In the latter case, which is of higher significance in PPP, the autocorrelation was weak and reached to 0.17 for the GPS L1 signal at the time lag of 0.2 s. On the other hand, Moschas and Stiros [34] demonstrated that coordinates derived from 100 Hz GNSS data are correlated for a lag up to 0.05 s. Furthermore, they also indicated the potential mitigation of the temporal correlation by the modification of the phase-locked loop (PLL) bandwidth, for example, from common 25 Hz up to 50–100 Hz. Taking into account these results and the fact that the frequency of the periodic oscillations in our experiment did not exceed 4 Hz, we assume that the temporal correlation distorts the displacements results to a lesser extent. As a result, we employed a simplified and well-known stochastic model without accounting for temporal correlation. Nevertheless, there is no doubt that this issue needs further investigations to improve stochastic modelling and hence reach the highest precision.

### 2.2. Filtration of PPP Coordinate Time Series for the Extraction of the High-Rate Dynamic Displacements

It is known that the high accuracy of the PPP solution (up 1 cm) may be reached after long (e.g., several hours) static solution [36]. However, in structural monitoring, the observational session may be much shorter. Moreover, the PPP-derived coordinate time series are still the subject of several un-modelled effects and residual biases, which may affect the estimated position. In this group, the most challenging to handle is the phase multipath. This effect may reach up to several centimeters, which importantly deteriorates the coordinate estimates. The impact of multipath can be mitigated with sidereal filtering, but such an approach requires a session of at least a few days, which is not always available. On the other hand, it should be remarked that these deteriorations are characterized with relatively long periods (several minutes and more) with respect to the analyzed dynamic displacements. Other long-term effects in the PPP time series may be related to the imprecision of satellite orbits and phase center variations, as well as residual impact of the tropospheric delay. Considering the latter, there is no doubt that estimated ZTD parameters represent only an average state of the troposphere, and thus the residuals at particular observables are basically expected. The last important factor, which propagates into the estimated coordinates, is the residual impact of satellites and receiver clocks. In this case, the short-term variations are rather expected. As a result of all of the unwanted errors, we cannot provide the mm-level position with regular PPP, even when employing multi-constellation and multi-frequency observations. However, according to the above, the most disturbing factors are expected to be long-term effects and can be effectively mitigated with high-pass filtering. Hence, such a process was applied to the coordinate time series to handle residual adverse effects and, consequently, to reach high-accuracy displacements. Owing to the known frequency of the simulated motion (3.8 Hz), it was decided to apply a high-pass Butterworth filter with cut-off period set to 2 s. This filter is one of the most commonly used tools in time series processing. Its response as a function of angular frequency (ω) can be written as follows:

(13) $$\left|H\left(j\omega \right)\right|=\frac{1}{\sqrt{1+{\left(\omega_{c}/\omega \right)}^{2n}}},$$

where $\omega_{c}$ corresponds to cut-off frequency and n is the order of filter. According to the equation, the filter has no ripple in pass band and is often labelled as maximally flat. The flat shape is realized at the expense of the wide transition region. This undesired effect can, however, be improved through the application of a higher order, which, in our case, was 6.

The high efficiency for seismic waveforms in GNSS estimates of this filter has been recently proven, for example, in the work of [37]. The selected threshold in the frequency domain allows the elimination of all the above indicated factors excluding short-term variations of coordinates related to the clocks residuals (mostly caused by less stable internal oscillator of receiver). The high-frequency component of these residuals cannot be simply separated from the noise of phase data, which consequently reduces the precision of PPP estimates. Nevertheless, we can assess the theoretical ratio of precisions for filtered time series derived from PPP and relative positioning. Neglecting the impact of residual clocks, the standard deviation of estimates is mainly driven by the phase data noise. If we assume the same precision at both frequencies used for IF combination, the ratio should be close to 1.5. As a result, we expect the precision of the filtered time series for PPP solution to be at the level of a few millimeters.

## 3. Experiment Design and Results

### 3.1. Shake-Table System

**Figure 1:**
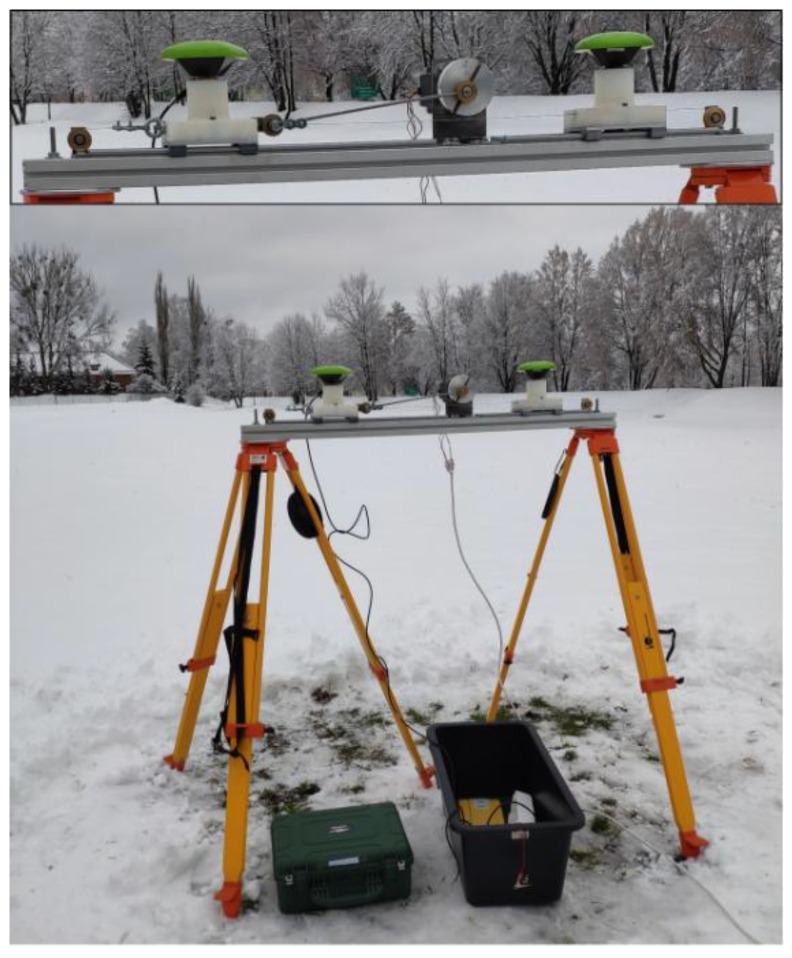
In-house developed portable shake-table system during data collection.

The experimental validation of the technology was based on the application of the prototype shake-table system. The system is responsible for the simulation of the dynamic displacements of the GNSS antenna in the horizontal plane with a high level of stability in terms of frequency and amplitude. This device is capable of setting a periodic motion in a single direction in a fully controllable manner. The advancement of the developed shake-table is the feasibility to set in motion two GNSS antennas, which, as a result, oscillate with the same amplitude and frequency. It is worth noting that the commercial systems mostly allow a single antenna application. The shake-table is fully portable, which makes it feasible to perform the field experiments at any place under real observation conditions and with a potential application of the existing GNSS reference networks (Figure 1).

**Figure 2:**
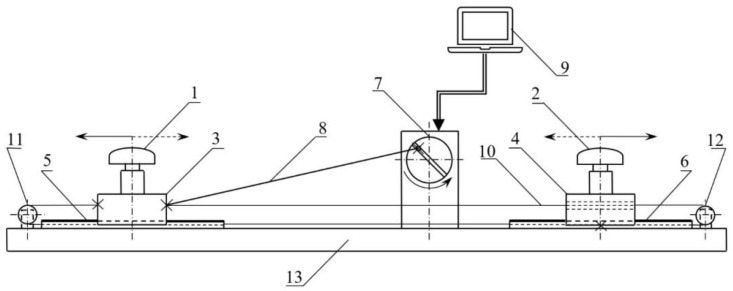
A scheme of the in-house developed shake-table system. The components of the shake-table system are as follows: (**1**) main GNSS antenna, (**2**) auxiliary GNSS antenna, (**3**) main trolley of GNSS antenna, (**4**) auxiliary trolley of GNSS antenna, (**5**) main GNSS antenna linear motion guide, (**6**) auxiliary GNSS antenna linear motion guide, (**7**) motion wheel with engine (stepper motor), (**8**) rigid driving tie, (**9**) control system built of a PC and dedicated drivers and software, (**10**) driving cord, (**11**) and (**12**) driving rolls, and (**13**) high-quality rigid frame.

The scheme of the shake-table system is presented in Figure 2. The system is built of several components, such as a stepper motor with the motion wheel, trolleys of the GNSS antennas, high-quality linear guides, a frame, and the control design environment system consisting of the dedicated software run under a PC. The developed device simulates the periodic motion with a frequency up to 25 Hz and amplitude fitting the range of 1–85 mm, with the precision of ±0.5 mm. Such a characteristic of the simulated dynamic displacements fully meets our requirements in terms of the validation of PPP for the application to GNSS-seismology and structural health monitoring. The detailed specification of the device is given in Table 2.

We may identify a number of benefits from the application of the shake-table to the validation of the technology aiming at dynamic displacement detection. The shake-table is especially advantageous for testing the response of high-rate multi-GNSS observations to known high-rate and low amplitude displacements. The characteristics of such an artificial motion of the GNSS antenna can be used as reference values for any monitoring system validation.

### 3.2. Experiment Design and Data Collection

The experiment was conducted under an unobstructed sky within the campus on the University of Warmia and Mazury in Olsztyn on 4 February 2019. In the experiment, we used GPS + Galileo + BDS observational data collected by Topcon Net-G5 multi-GNSS receiver with an interval of 100 Hz. Additionally, a 4 km distant Trimble NETR9 receiver was used to collect data for 50 Hz RTK, which served as a benchmark solution. Both sites were occupied by geodetic antennas: JAV_GRANT-G3T and TRM59800.00, respectively. Data collection started at 11:00 UTC time and lasted about 45 min. The session was divided into sub-sessions with different characteristics of the simulated oscillations. During first 10 min, the antenna was static; hence, this period was used for initial evaluation of the noise in the high-rate solution and for the determination of the initial antenna position. In the next step, we simulated horizontal oscillations of the antenna. The periodic oscillations were characterized with the frequency of 3.80 Hz and three amplitudes equal to 9 mm, 6 mm, and 1.5 mm, respectively. The selected frequency of the antenna oscillation fits the range of the frequencies that are common for the engineering structure’s vibrations induced by geohazard phenomena and other external forces. Each session with different characteristics of the motion lasted approximately 10 min.

**Figure 3:**
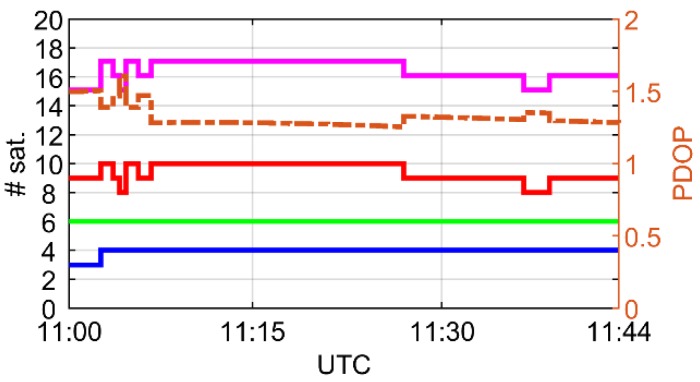
Number of satellites and corresponding PDOP during the data collection time span. Solid blue, red, green, and magenta lines correspond to BDS, GPS, Galileo, and total number of satellites, respectively. Dotted line visualizes PDOP of multi-constellation solution.

In Figure 3, we depict the number of tracked satellites versus PDOP of the combined solution. From the figure, we can learn that, as expected, the most of the tracked satellites were of GPS constellation. In this case, we could acquire the signals from up to 10 satellites. This number was equal to 6 and 4 satellites in the case of the Galileo and BDS systems, respectively. Looking at the PDOP values, we may conclude on good observation conditions during the experimental session. As the number of tracked satellites and PDOP did not vary significantly during the data collection time span, this session can be considered as homogenous with regard to the observation conditions.

### 3.3. Initial Evaluation of the High-Rate PPP Displacement Time Series Noise

In the first step, we aim to evaluate the noise of the high-rate kinematic PPP solution. For this purpose, we used the coordinate time series corresponding to the period when the GNSS antenna was motionless (first 10 min of the observing session). In this case, we consider any deviation from the reference position as the coordinate residual. The multi-constellation PPP solution was referred to the single constellation PPP, as well as to benchmark solutions obtained with the kinematic relative positioning: GPS and multi-constellation. At this point, we should note that the employed coordinate time series were filtrated with a high-pass Butterworth filter with a cut-off period set to 2 s. In this way, we disposed of any residual long-term errors. Such a process is justified because we aim to retrieve high-rate coordinate changes.

**Figure 4:**
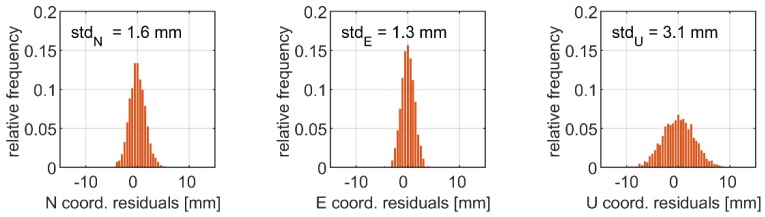
Histograms of coordinate residuals obtained with real-time kinematics (RTK) G + E + C.

**Figure 5:**
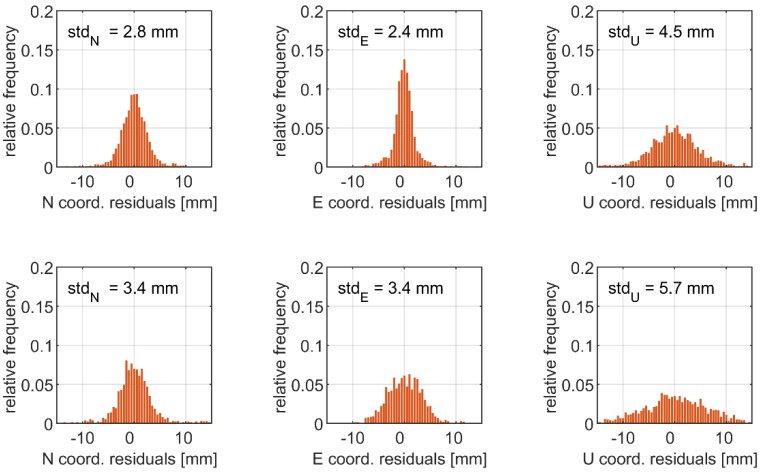
Histograms of coordinate residuals obtained with precise point positioning (PPP) G (**first row**) and PPP G + E + C (**second row**).

In Figure 4, the histograms of the coordinate residuals derived from the multi-constellation relative mode solution are given. These results serve as the benchmarks to PPP. Figure 5 shows corresponding values for GPS and multi-constellation PPP solutions. As the statistics characterising the noise, we used standard deviations (*std*), which may be found in Table 3. The standard deviations of the PPP coordinate residuals fitted the ranges of 2.4–3.4 mm and 4.5–5.7 mm for the horizontal and height components, respectively. We noticed slight differences in the coordinate noise between the single-system and multi-constellation solutions. This is reflected in *std* of the multi-constellation PPP coordinate time series, which was higher from 0.6 mm up to 1.2 mm with respect to the single-system solution. The results may indicate varied precision of the phase measurements of analysed GNSS systems. In our case, significant differences in the accuracy of satellite orbits and clocks between the constellations reported in a previous study [38] are of lower impact, because their influence is mitigated by the high-pass filtering. The unwanted effects in the coordinate time series induced by the inaccuracies of satellite products are characterized by a low-frequency trend, which may be easily eliminated when retrieving high-frequency dynamic displacements with filtering.

The precision of the PPP coordinate time series was slightly lower with respect to the relative mode solution. In the latter case, the multi-system solution resulted in *std* of N, E, and U components reaching the level of 1.6 mm, 1.3 mm, and 3.1 mm, respectively (Table 3). Slightly higher precision of the relative mode solution is expected, recalling that a short baseline was employed (4 km) and that uncombined observables were applied. Nonetheless, the results confirmed a low noise of the high-frequency coordinate time series for both the relative and absolute modes.

We may set up a hypothesis that, in the case of the applications of the highest precision (at mm-level), multi-GNSS signals do not significantly improve the precision of the solution. This statement holds true, providing an acceptable number of tracked satellites. However, much more reliable verification of this hypothesis may be performed in the scenario with simulated high-rate displacements of the GNSS antenna, which is presented in the next section. Nonetheless, a number of studies clearly showed that multi-GNSS signals contribute to the reduction of the time to fix and of the mean position residuals [39,40,41,42]. In our case, these indicators are of lower importance because of support from filtering.

### 3.4. Applicability of High-Rate PPP to the Detection of Dynamic Displacements

The applicability of the enhanced PPP for the determination of small-scale dynamic displacements was evaluated by referring the parameters characterising periodic oscillations of the GNSS antenna, namely frequency (F) and amplitude (A), to the true values simulated by the shake-table system. Additionally, a short baseline relative solution was employed as an additional benchmark. The RTK solution was obtained with 50 Hz data, owing to the observation interval capabilities of the receiver at the reference station. In order to extract small-scale high rate displacements from the coordinate time series, we used a high-pass Butterworth filter with the cut-off period set to 2 s. In this way, we handled the low-frequency effects in the PPP coordinate time series, which are mainly caused by residual GNSS positioning errors. As stated before, the shake-table simulated horizontal single-direction periodical oscillations of the frequency of 3.80 Hz and three selected amplitudes of 9 mm, 6 mm, and 1.5 mm, respectively.

**Figure 6:**
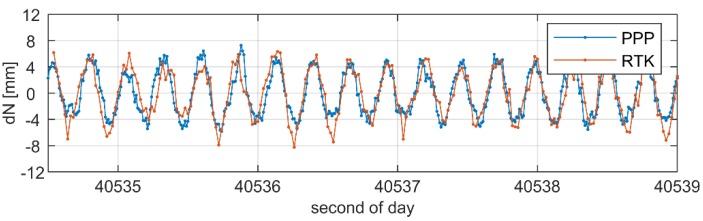
Displacement time series of the N–S component obtained from multi-GNSS 100 Hz PPP and 50 Hz RTK solutions during the session with simulated oscillations of 9 mm amplitude.

**Figure 7:**
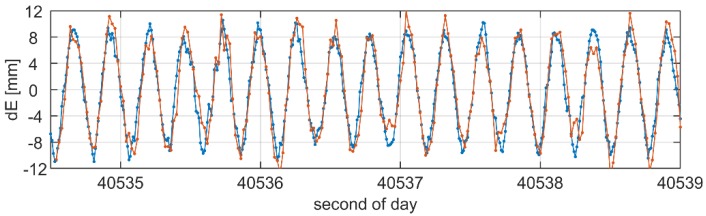
Displacement time series of the W–E component obtained from multi-GNSS 100 Hz PPP and 50 Hz RTK solutions during the session with simulated oscillations of 9 mm amplitude.

**Figure 8:**
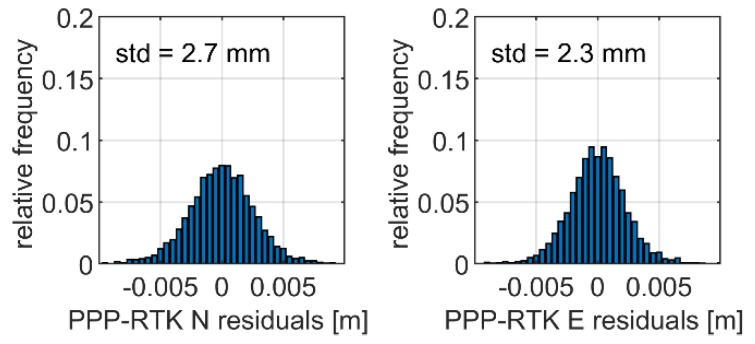
Histograms of differences between multi-GNSS PPP and RTK displacements given for the N (**left panel**) and E (**right panel**) components.

Figure 6 and Figure 7 present a focus on the displacement time series obtained from the multi-GNSS PPP and multi-GNSS RTK solutions during the period when the oscillations were characterized with the amplitude of 9 mm and the frequency of 3.80 Hz. The time series are given separately for the E–W (Figure 6) and N–S components (Figure 7). From the figures, we may clearly read the periodic oscillations of the antenna position. Even a cursory analysis of the results points to high agreement between the PPP and RTK solutions. The detailed results of the analyses are illustrated in Figure 8, where the histograms of the differences in displacement domain between both solution are given. The histograms do not reveal any systematic biases between both solutions, which is confirmed by a lack of the shift. Looking at the statistics, we may conclude on the consistency of both solutions, as the standard deviation of the PPP–RTK differences reached only 2.7 mm and 2.3 mm for the N and E components, respectively. In our opinion, such outcomes confirm high agreement of PPP with the RTK results. The standard deviations are low and correspond to the noise of the position solution. 50 Hz RTK solutions during the session with simulated oscillations of 9 mm amplitude.

**Figure 9:**
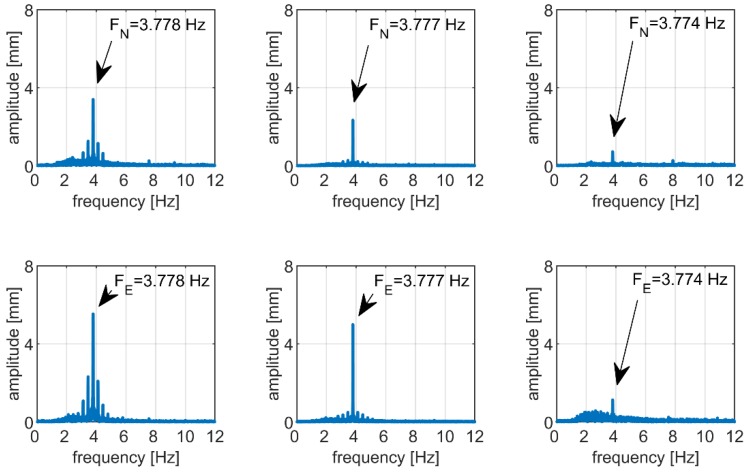
Frequency responses extracted from GNSS–PPP displacement time series when simulating an antenna oscillation of 3.80 Hz frequency and amplitudes of 9 mm (**left panel**), 6 mm (**middle panel**), and 1.5 mm (**right panel**). Top and bottom panels correspond to the north and east coordinate component, respectively.

As both methods use the same receiver and GNSS signals, much more reliable validation of the PPP results may be performed by taking advantage of independent technique, which, in this case, is the comparison to benchmark values of the frequency and amplitude simulated by the shake-table. Table 4, Table 5 and Table 6 provide the frequency responses obtained from Fourier transformation (FT) for three scenarios of simulation, that is, using an amplitude of 9, 6, and 1.5 mm, respectively. Figure 9 visualizes corresponding FT spectrum. In all cases, FT allowed the detection of the dominant frequency in an agreement with each other and with the designed value, which was equal to 3.800 Hz. The maximal difference of the peak frequency between PPP and the benchmark value did not exceed 0.026 Hz, as the detected frequencies fitted the range from 3.774 to 3.778 Hz.

An analysis of the amplitude detected from the GNSS–PPP solution provides us with an evaluation of precision in a displacement domain. The differences between the benchmark amplitudes of the antenna oscillations and the mean amplitudes obtained from PPP were lower than 1 mm in the case of 9 mm and 6 mm simulated amplitudes (Table 4 and Table 5). We consider such results to be of high precision. Slightly higher residuals were obtained in the case of simulated oscillations characterized with 1.5 mm amplitude (Table 6). In this case, the mean amplitudes detected with PPP were in the range of 2.0–2.8 mm depending on the processing strategy, GPS or multi-constellation, respectively. We should, however, note that such residuals correspond to the level of the phase observational noise.

The results of the single-system solution versus multi-constellation are in line with the results of the coordinate time series noise given in Section 3.3, as we did not discover a benefit from multi-constellation signals. Specifically, we detected exactly the same mean amplitudes for GPS and multi-GNSS solutions when simulating the periodic oscillations with the amplitudes of 9 mm and 6 mm. Slight differences were only detectable when the oscillations were simulated with 1.5 mm amplitude. In this case, we isolated 2.0 mm amplitude from the GPS–PPP results and 2.8 mm from GNSS–PPP, respectively. We believe that a slightly higher noise of the multi-GNSS solution had the dominating impact on such results.

The experiment results justified a high-applicability of high-rate PPP for the detection of structural vibration in SHM. Similar conclusions were also raised by Yigit and Gurlek [43] and Tang et al. [44]; however, as recent studies showed, progress may still be made, for example, by isolation and modelling of the multipath effect [45].

## 4. Conclusions

In this paper, we presented the enhanced kinematic PPP aiming at high-rate small-scale displacement detection. The method’s validation was based on the field experiment performed with the use of the prototype shake-table. The device was responsible for the simulation of the periodic single-direction oscillations of the antenna with the frequency of 3.80 Hz and the amplitudes of 9 mm, 6 mm, and 1.5 mm, respectively. The validation of the method was performed twofold, by the comparison with the high-rate RTK solution as well as by the reference of the parameters characterising the detected oscillations, namely frequency and amplitude, to the benchmark values that were set up in the simulation scenario. The results confirmed a high applicability of the method. We were able to detect the frequency and amplitude of the simulated displacements with a high level of accuracy. Specifically, the differences between the PPP-derived and true amplitude of the simulated displacements were in the range of 0.5–1.3 mm, whereas the difference between the detected and benchmark frequencies did not exceed 0.026 Hz. The experiment results also showed a high agreement between relative and absolute GNSS solutions in the displacement domain. The standard deviations characterising the displacement residuals between the PPP and RTK solutions were equal to only 2.7 mm and 2.3 mm for the N and E components, respectively.

The analysis revealed that, in the case of the presented processing strategy, which was supported with the filtering, we may not expect much benefit from multi-constellation signals. We believe that at that level of precision, the dominant impact on the final accuracy is driven by phase observable noise. This statement holds true, provided that the receiver tracks an adequate number of satellites. On the other hand, it is clear that multi-GNSS signals contribute to the reduction of the time to fix and of the mean position residuals.

Our further investigations will focus on handling of time correlations and isolating the multipath effect in high-rate GNSS data processing. In this way, we will be able to fully benefit from high-rate observables, and hence improve the precision of the solution.

## Figures and Tables

**Table 1:** Details of the processing strategy applied to precise point positioning (PPP).

Option	Setting
Signals	Phase and code GPS L1/L2, BDS B1/B2, and Galileo E1/E5a
Sampling rate	0.01 s (100 Hz)
Observable combination	Undifferenced ionosphere-free linear combination (IF-LC)
Weighting scheme	Elevation-dependent weighting
Elevation cutoff angle	10°
Troposphere delay modelling	Estimation of the residual ZTD, a priori modified Hopfield model + global mapping function
Ionosphere delay modelling	First order of ionospheric delay eliminated taking advantage of IF-LC
Type of the solution	Float kinematic
Stochastic modelling: a priori standard deviation of observations	0.3 m/0.003 m for code/phase of GPS and Galileo signals, respectively; BDS signals down-weighted by the value of 20%.
Satellite orbits and clocks	Precise CODE (interval: orbits 5 min; clocks 30 s)
Parameter estimation method	Sequential least squares with a priori parameter constraining and equivalent elimination

**Table 2:** Specification of in-house developed shake-table system.

Feature	Value
Dimensions	1.34 m length, 0.23 width, 0.18 m height (without antenna)
Total mass	10 kg
Number of GNSS antennas that can be set in motion	2
Maximum load weight	4 kg
Number of motion axes	single
Characteristics of the motion	Linear, periodical in selected horizontal direction
Maximum frequency of the oscillations	Up to 25 Hz
Amplitude range	1–85 mm
Precision of dedicated amplitude	−0.5 mm
Control system	dedicated software running under a PC
Engine	Stepper motor

**Table 3:** Evaluation of the coordinate time series noise—statistics of coordinate residuals. RTK, real-time kinematics.

Type of Solution	std_N_ [mm]	std_E_ [mm]	std_U_ [mm]
RTK G + E + C	1.6	1.3	3.1
PPP G	2.8	2.4	4.5
PPP G + E + C	3.4	3.4	5.7

**Table 4:** Frequency response extracted from N, E displacement time series when simulating an antenna oscillation of 9 mm amplitude.

	Detected Values			Benchmark Values	
	F_N_ [Hz]	F_E_ [Hz]	A [mm]	F [Hz]	A [mm]
RTK G + E + C	3.775	3.775	9.1	3.800	9.0
PPP G	3.778	3.778	10.0		
PPP G + E + C	3.778	3.778	10.0		

**Table 5:** Frequency response extracted from N, E displacement time series when simulating an antenna oscillation of 6 mm amplitude.

	Detected Values			Benchmark Values	
	F_N_ [Hz]	F_E_ [Hz]	A [mm]	F [Hz]	A [mm]
RTK G + E + C	3.777	3.777	7.6	3.800	6.0
PPP G	3.777	3.777	6.9		
PPP G + E + C	3.777	3.777	6.9		

**Table 6:** Frequency response extracted from N, E displacement time series when simulating an antenna oscillation of 1.5 mm amplitude.

	Detected Values			Benchmark Values	
	F_N_ [Hz]	F_E_ [Hz]	A [mm]	F [Hz]	A [mm]
RTK G + E + C	3.773	3.773	3.1	3.800	1.5
PPP G	3.774	3.774	2.0		
PPP G + E + C	3.774	3.774	2.8		
